# A comparison of psychiatric diagnoses among HIV-infected prisoners receiving combination antiretroviral therapy and transitioning to the community

**DOI:** 10.1186/s40352-014-0011-1

**Published:** 2014-10-29

**Authors:** Angela Di Paola, Frederick L Altice, Mary Lindsay Powell, Robert L Trestman, Sandra A Springer

**Affiliations:** 1 Yale University School of Medicine, Department of Internal Medicine, Section of Infectious Diseases, AIDS Program, 135 College Street, Suite 323, New Haven, CT 06519 USA; 2 Yale University School of Public Health, Division of Epidemiology of Microbial Diseases, 60 College Street, New Haven, CT 06519 USA; 3 University of Connecticut Health Center, Correctional Managed Health Care, 263 Farmington Avenue, Farmington, CT 06030 USA

**Keywords:** Psychiatric disorders, Prisoners, Criminal justice system, HIV, Substance abuse

## Abstract

**Background:**

The criminal justice system (CJS), specifically prisons and jails, is ideally suited for uniform screening of psychiatric (PD) and substance use disorders (SUDs) among people living with HIV/AIDS (PLWHA), who are concentrated in these settings. By accurately diagnosing PDs and SUDs in these controlled settings, treatment can be initiated and contribute to improved continuity of care upon release. In the context of PLWHA, it may also improve combination antiretroviral treatment (cART) adherence, and reduce HIV transmission risk behaviors.

**Methods:**

A retrospective data analysis was conducted by creating a cohort of PLWHA transitioning to the community from prison or jail enrolled who were enrolled in a controlled trial of directly administered antiretroviral (DAART). Participants were systematically assessed for PDs and SUDs using the Mini International Neuropsychiatric Interview (MINI), a standardized psychiatric assessment tool, and compared to diagnoses documented within the correctional medical record.

**Results:**

Findings confirm a high prevalence of Axis I PDs (47.4%) and SUDs (67.1%) in PLWHA even after prolonged abstinence from alcohol and drugs. Although prevalence of PDs and SUDs were high in the medical record, there was fair to poor agreement among PDs using the MINI, making evident the potential benefit of more objective and concurrent PD assessments to guide treatment.

**Conclusions:**

Additional PD diagnoses may be detected in PLWHA in CJS using supplementary and objective screening tools. By identifying and treating PDs and SUDs in the CJS, care may be improved and may ultimately contribute to healthier outcomes after community release if patients are effectively transitioned.

**Electronic supplementary material:**

The online version of this article (doi:10.1186/s40352-014-0011-1) contains supplementary material, which is available to authorized users.

## Background

One in 100 adults in the United States (U.S.) is incarcerated, with one in 31 under community supervision in parole or probation (Pew Center on the States [2009]). In the incarcerated population, HIV and psychiatric disorders (PDs) are concentrated and syndemic, with each negatively impacting the outcome of treatment and prevention efforts. Axis I PDs and substance use disorders (SUDs) are concentrated among prisoners within the criminal justice system (CJS), being two-fold and 9-fold greater than found in the general population (James et al. [2006]; Substance Abuse and Mental Health Services Administration [2011]), respectively, as defined by the 4^th^ Diagnostic and Statistical Manual of Mental Disorders (DSM-IV) (American Psychiatric Association. Task Force on, D.-I [2000]); similarly, the prevalence of people living with HIV/AIDS (PLWHA) is 3- to 4-fold greater within CJS than the general population, respectively (Spaulding et al. [2009]; Asner-Self et al. [2006]; Diamond et al. [2001]; Baillargeon et al. [2003]). Prison-involved PLWHA have higher rates of PDs than those without HIV, and PDs are higher among this population compared to those who are not incarcerated (Rich et al. [2011]; Altice et al. [2010]).

In the absence of HIV infection, PDs often remain undiagnosed and untreated due to a myriad of reasons (Christiana et al. [2000]; Holden et al. [2012]; Draine et al. [2002]; Link et al. [1997]; Hines-Martin et al. [2003]). It is estimated that 3 in every 5 persons with a mood, anxiety or SUD do not seek professional help in the first year of symptoms, moreover, it is common not to seek help for up to 10 years after symptoms begin (Christiana et al. [2000]). Furthermore, African Americans, who are disproportionately concentrated in the CJS, are less likely than Caucasians to seek help for PDs (Holden et al. [2012]). Prior treatment of PDs and psychiatric symptoms may not be reported to healthcare professionals to avoid the stigma associated with receiving a PD diagnosis (Link et al. [1997]). Additionally, in the presence of active substance use, inadequate diagnosis and treatment of PDs in community settings unwittingly contribute to the overburdened CJS (Draine et al. [2002]), which struggles with insufficient resources, yet these structured settings makes them suitable for standardized screening and treatment algorithms (Finkelstein et al. [2005]; Maruschak and Beavers [2009]; Kamath et al. [2013]). Consequences of undiagnosed and under-treated PDs among PLWHA can result in poor treatment outcomes for those transitioning to the community where the highly structured prison setting is discontinued, resulting in poor access and adherence to combination antiretroviral therapy (cART) (Springer et al. [2012]; Meyer et al. [2011]), suboptimal viral suppression (Uldall et al. [2004]; Springer et al. [2004]; Meyer et al. [2011]; Meyer et al. [2014]) and increased HIV risk-taking behaviors (Goforth and Fernandez [2011]; Buckingham et al. [2013]), repeated incarcerations (Baillargeon et al. [2009]; Baillargeon et al. [2010a]; Baillargeon et al. [2010b]), relapse to drug and alcohol use (Krishnan et al. [2013]), decreased retention in HIV care (Althoff et al. [2013]), a high frequency of emergency department visits (Meyer et al. [2012], [2013]), and a higher risk of death (DeLorenze et al. [2010]). Thus, correctly identifying and treating PDs among HIV-infected prisoners prior to release is crucial for effective transitional care of PLWHA who re-enter the community (Springer et al. [2011]).

Given the negative consequences of PDs on HIV treatment outcomes, we sought to investigate the prevalence of DSM-IV PDs among a cohort of PLWHA prescribed cART who were transitioning from prison to the community, assessed during a prolonged period of likely abstinence from drugs and alcohol, and whether they were diagnosed and treated.

## Methods

### Participants

**Figure 1:**
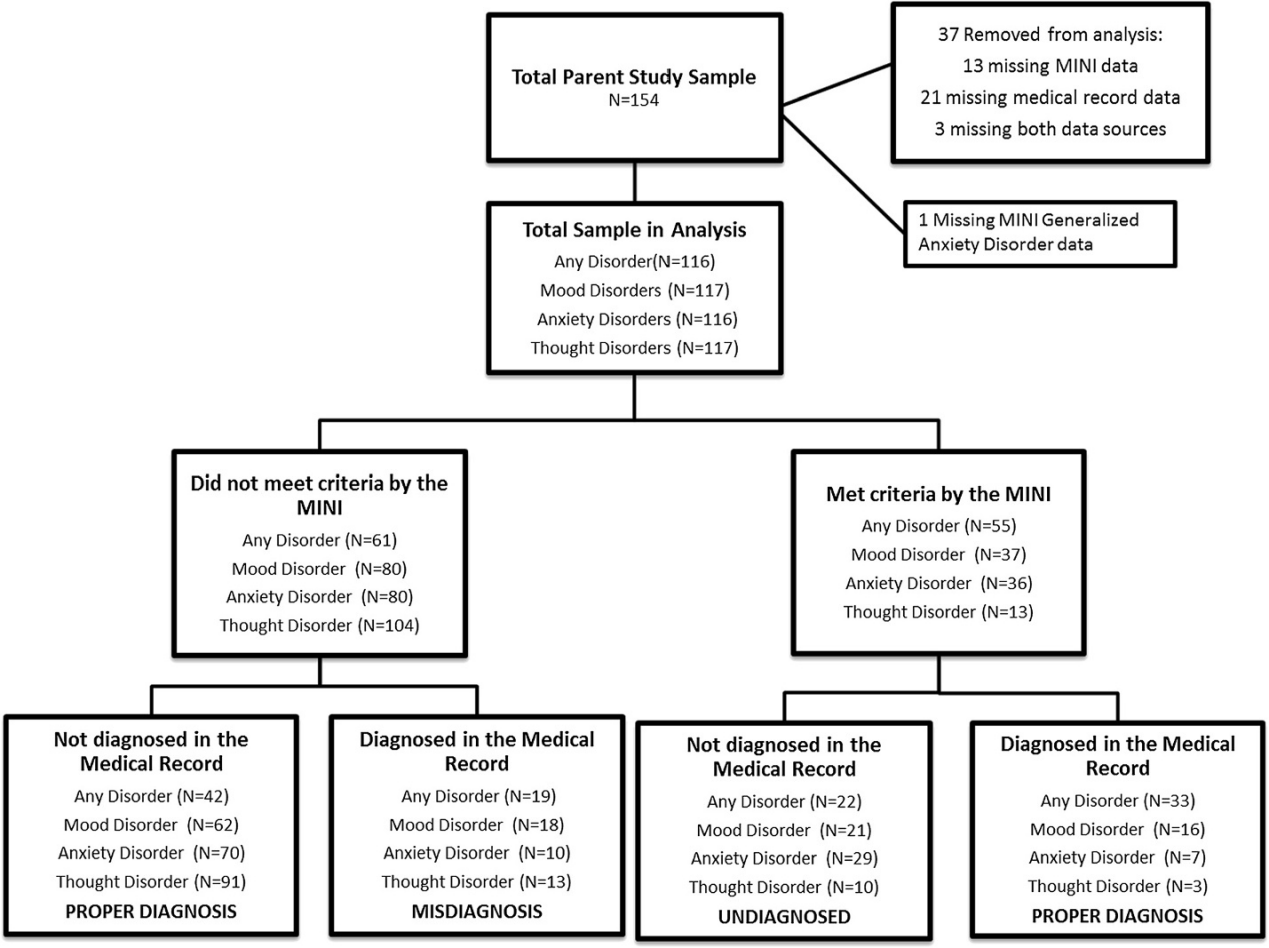
**Participant consort flow diagram.**

This was a retrospective data analysis of a previously described prospective randomized controlled trial (RCT) of directly administered antiretroviral treatment (DAART) versus self-administered treatment (SAT) among HIV-infected prisoners prescribed cART and with a pre-incarceration history of heroin or cocaine use in the 6 months prior to incarceration who were transitioning to the community. The details of the trial have been described previously (Altice et al. [2011]; Saber-Tehrani et al. [2012]), but briefly, 154 PLWHA participants prescribed cART, age ≥18 years, and within 90 days of release and returning to two areas in Connecticut (New Haven or Hartford) upon release (F.L. Altice et al. [2011]; Saber-Tehrani et al. [2012]) were recruited from 2004 through 2009. All participants underwent a baseline assessment that included demographic characteristics, drug use and addiction severity using the Addiction Severity Index (ASI) (McLellan et al. [1992]; Rikoon et al. [2006]; McLellan et al. [2006]), and the Mini International Neuropsychiatric Interview (MINI) (Sheehan et al. [1997]; D. V. Sheehan et al. [1998b]; Amorim et al. [1998]; Lecrubier et al. [1997]) assessed DSM-IV PDs in the absence of drug or alcohol use (>7 months) during incarceration and prior to community release. Of the 154 recruited, 37 participants were excluded due to missing data, either the clinical chart review (N = 21), the MINI (N = 13) or both (N = 3) were missing, resulting in 117 participants included in the final analysis (Figure 1). The parent study's randomized intervention was conducted after the baseline assessments were obtained, therefore the intervention did not impact this particular analysis.

### Protections of human subjects

The parent study of DAART versus SAT (Altice et al. [2011]; Saber-Tehrani et al. [2012]) was approved by Yale University Human Investigation Committee and Connecticut Department of Correction Research Advisory Committee. Due to the inclusion of prisoners in this study additional assurances were provided by the Office of Human Research Protections and a Certificate of Confidentially was obtained.

### Measures of psychiatric disorders

After extensive training by developers of the MINI 5.0 computerized version, trained researchers administered the MINI to participants while they were abstinent from alcohol or drugs. It is a short diagnostic interview with excellent reliability and validity for DSM-IV diagnoses (Sheehan et al. [1997]; D. V. Sheehan et al. [1998b]; Amorim et al. [1998]; Lecrubier et al. [1997]) and validated to the Structured Clinical Interview (SCID-P) (Spitzer et al. [1992]), and the Composite International Diagnostics (CIDI) (Robins et al. [1988]) for International Statistical Classification of Disease (ICD-10) (D. Sheehan et al. [1997]; Sheehan et al. [1998b]; Amorim et al. [1998]; Lecrubier et al. [1997]) with high construct validity and internal and external consistency. For this analysis, current (the past 12 months) and lifetime symptom diagnoses from selected MINI 5.0 modules, were used as the "gold standard" (Sheehan et al. [1997]; Sheehan et al. [1998b]; Amorim et al. [1998]; Lecrubier et al. [1997]).

For care and treatment, the Connecticut Department of Correction (CTDOC) contracts the University of Connecticut Health Center Correctional Managed Health Care to follow a standardized Mental Health Policy (State of CT Dept of Correction, [2008]), which outlines the screening and evaluation process. Intake screenings are conducted by a licensed social worker or registered nurse within 24 hours of referrals for: all first time incarcerations, those discharged from a psychiatric facility within 30 days, those who display or indicate suicidal ideation within 30 days of incarceration, inmates that are indicated by the court or concerned party to have mental health concerns, or inmates with either self or concerned party reports of a history of suicide attempts or plans within three years. Additionally, self-referrals or referrals from concerned parties are evaluated within 72 hours (State of CT Dept of Correction [2008]). Through a class action lawsuit, additional mental health screening is mandated for prisoners with HIV. Inmate classification scores are used by the CTDOC to quantify the severity of health care needs related to underlying PDs ranging from 1 (no issues) to 5 (24-hour nursing needs). (These classification scores are available at: http://www.ct.gov/doc/lib/doc/PDF/PDFReport/ClassificationManualLibraryCopy.pdf). Although no standardized screening tools are used, all HIV-infected prisoners are assessed further by a licensed professional. After screening, triage to other professionals for further assessment and treatment is performed when indicated and the diagnoses noted within the clinical record are used as the basis within prison. Medical records reflect all diagnoses from community medical records and those made within the CTDOC and are noted as active or inactive.

### Additional measures

Baseline characteristics included gender, age, ethnicity, housing status, education level, randomization group (DAART vs. SAT), drug use assessment of opioid or cocaine use, hazardous drinking, participation in pre-incarceration opioid substitution therapy and employment status. All SUDs using the MINI or Alcohol Use Disorder Identification Test (AUDIT) (Saunders et al. [1993]; Barbor [2001]) assessed the 12-month time period before the incarceration. Opioid and cocaine use disorders were determined by using DSM-IV criteria for opioid or cocaine dependence and abuse; alcohol use disorders were assessed by the AUDIT, scores of eight or greater for men or four or greater for women were used to define an alcohol use disorder (Bradley et al. [1998]; National Institute on Alcohol Abuse and Alcoholism [2005]). Score for opioid, cocaine and alcohol use disorders were coded dichotomously. Additional drug use and addiction and psychiatric symptom severity was ascertained using the ASI version 5 (McLellan et al. [1992]) as well as psychiatric symptom severity for the 30 days prior to incarceration. The ASI composite drug (>0.16), alcohol (>0.17) and psychiatric (>0.22) scores were reported as dichotomous variables using cut-offs scores shown to have high levels of sensitivity and specificity for DSM-IV diagnoses (Rikoon et al. [2006]; Calsyn et al. [2004]).

Additional baseline characteristics collected from the participant's prison medical record included prescription information for psychiatric medications, incarceration dates, antiretroviral (cART) medications, and pre-release HIV treatment status consisting of: HIV RNA viral load (VL), and CD4 lymphocyte counts (CD4).

### Analysis

Bivariate logistic regression analyses of the different psychiatric diagnosis by the MINI or medical record were used to assess the differences in baseline demographic characteristics. Statistical significance was defined at p < 0.05 for all associations. Levels of agreement between the MINI and medical record were calculated using Cohen's Kappa (Cohen [1960]). Statistical analysis was conducted using SPSS Version 19 (SPSS Inc., Chicago, IL, USA).

MINI diagnoses were compared to diagnoses recorded in the correctional medical record using logistic regression. Diagnoses from both the MINI and medical record were categorized into three major types of Axis I disorders: mood, anxiety and thought disorders. Mood disorders included current symptom diagnoses within past 12 months of major depression and bipolar disorders; anxiety disorders included current (past 12 month) symptom diagnosis of panic disorder, agoraphobia, obsessive compulsive disorder (OCD), post-traumatic stress disorder (PTSD), generalized anxiety disorder (GAD), and lifetime symptom diagnosis for panic disorder; and thought disorders included psychotic disorder with and without schizophrenic features for current and lifetime symptom diagnoses.

## Results

### Demographic characteristics

Baseline characteristics (Table 1) demonstrated that participants were primarily Black (53.8%) or Hispanic (32.5%) men (82.1%) in their mid-40s. Over half (51.3%) anticipated unstable housing (with a family member or friend temporarily, transitional housing, drug treatment facility, or not knowing) or being homeless (24.2%: in a homeless shelter or on the street) upon prison release. Clinically, 29.9% were prescribed psychiatric medications and all were prescribed cART (most were prescribed a protease inhibitor-based cART regimen [64.3%]), the majority (78.6%) achieved viral suppression (VL < 400 copies/mL) prior to release and had a mean CD4 of 403.6 cells/mL.

**Table 1:** **Baseline Characteristics**

Baseline characteristics	Total sample
	(N=117)
Gender	
Male	96 (82.1%)
Female	21 (17.9%)
Mean Age, years (SD)	45.4 (±6.9)
Ethnicity	
White	16 (13.7%)
African-American	63 (53.8%)
Hispanic	38 (32.5%)
Anticipated Housing	
Unstable Housing	60 (51.3%)
Homeless	29 (24.8%)
Stable Housing	21 (17.9%)
Opioid Use Disorder*	43 (36.8%)
Cocaine Use Disorder*	54 (46.2%)
Hazardous Drinking (AUDIT**)	50 (42.7%)
Viral Load (Baseline)	
< 400 copies/mL	92 (78.6%)
< 50 copies/mL	66 (56.4%)
Mean Log HIV-1 RNA Level, cells/mL (SD)	2.300 (±1.02)
CD4+ lymphocytes count, cells/mL (SD)	403.61 (±244.4)
Addiction Severity Index Composite Scores	
Psychiatric Composite Score (n=112)	
Mean Severity Score (SD)	0.254 (±0.25)
High Severity (>.22)	52 (46.4%)
Drug Use Composite Score (n=113)	
Mean Severity Score (SD)	0.085 (±0.08)
High Severity (>.16)	15 (13.3%)
Alcohol Use Composite Score (n=112)	
Mean Severity Score (SD)	0.055 (±0.11)
High Severity (>.17)	8 (7.1%)
Prescribed Psychiatric Medications	35 (29.9%)
Anti-Depressants	22 (18.8%)
Anti-Psychotics	2 (1.7%)
Mood Stabilizers	1 (0.9%)
Multiple Medications	16 (13.7%)
Any Psychiatric Disorder (n=116)*	55 (47.4%)
Any Mood Disorder*	37 (31.6%)
Any Anxiety Disorder (n=116)*	36 (31.0%)
Any Thought Disorder*	13 (11.1%)

SD=standard deviation.

*Criteria for diagnosis defined by the Mini International Neuropsychiatric Interview (MINI). Substance use disorders combine abuse and dependence diagnosis criteria.

**Hazardous Drinking defined by the Alcohol Use Disorders Identification Test (AUDIT), scores ≥8 for men, ≥4 for women.

### Prevalence of psychiatric disorders

Table 2 depicts the agreement of PD diagnoses made by the MINI and medical record. Both the MINI (47.4%) and medical record (44.8%) confirmed high prevalence of having any PD; concordance between the two was considered fair. Using the MINI, the prevalence of mood disorders was 31.6% overall, with 12.8% of the full sample meeting criteria for major depressive disorder and 18.8% for bipolar disorder. Similarly, 31.0% met criteria for having an anxiety disorder, with 14.7% having panic disorder, 12.0% OCD, 6.9% PTSD and 11.2% GAD. Last, 11.1% of the sample met criteria of having a thought disorder, with 7.7% having a current psychotic disorder and an additional 3.4% met criteria for lifetime psychotic disorder.

**Table 2:** **Diagnoses Frequencies by Measure and Kappa Values**

Psychiatric Disorder (N=117)	MINI diagnosis	Medical record diagnosis	No diagnosis by either measures	Diagnoses captured by both measures	Kappa level of agreement
**Any diagnosis (n=116)**	55 (47.4%)	52 (44.8%)	42 (36.2%)	33 (28.4%)	0.294 Fair
**Mood disorder**	37 (31.6%)	34 (29.1%)	62 (53.0%)	16 (29.1%)	0.212 Fair
Major Depressive Disorder	15 (12.8%)	26 (22.2%)	82 (70.1%)	6 (24%)	0.155 Poor
Bipolar Disorder	22 (18.8%)	15 (12.8%)	82 (70.1%)	2 (5.7%)	-0.052 Poor
**Anxiety disorder (n=116)**	36 (31.0%)	17 (14.7%)	70 (59.8%)	7 (15.2%)	0.081 Poor
Panic Disorder	17 (14.7%)	0 (0.0%)	99 (85.3%)	0 (0.0%)	0.000 Poor
Obsessive Compulsive					
Disorder	14 (12.0%)	0 (0.0%)	103 (88.0%)	0 (0.0%)	0.000 Poor
Post-Traumatic Stress					
Disorder	8 (6.9%)	13 (11.2%)	97 (82.9%)	1 (5%)	0.012 Poor
Generalized Anxiety	13 (11.2%)	5 (4.3%)	99 (85.3%)	1 (5.9%)	0.052 Poor
**Thought disorder**	13 (11.1%)	16 (13.7%)	91 (77.8%)	3 (11.5%)	0.096 Poor

While the prevalence of mood and thought disorders was similar using the MINI and the medical record, anxiety disorders were higher using the MINI (31.0% versus 14.7%, p = 0.33). Among the subgroups of anxiety disorders, the MINI diagnosed significantly more panic disorders (14.7% vs. 0%, p < 0.001), and OCD (12.0% vs. 0%, p < 0.001) when compared to diagnoses documented in the medical record.

### Agreement of Psychiatric Disorder diagnoses

Despite that both the MINI and the medical record review confirmed high levels of PD, the level of agreement between the two was at best, fair, and mostly poor. The highest (fair) level of agreement (kappa = 0.289) was found for having any PD diagnosis suggesting being correctly diagnosed with a PD. When we further examined the levels of agreement for specific diagnoses, however, levels of agreement decreased. Levels of agreement for those diagnosed with a mood disorder supported a fair level of agreement (kappa = 0.212), however, there was considerable disagreement for major depressive (kappa = 0.155) and bipolar (kappa = –0.052) disorders, respectively.

Levels of agreement for any anxiety disorder (kappa = 0.081) or thought disorder (kappa = 0.096) were poor and remained similarly low for each specific diagnosis among the various anxiety and thought disorders examined.

### Co-morbidity of substance use disorders

Overall, SUDs were highly prevalent along with the various PDs (Table 3). Using the MINI criteria, the prevalence of any SUD among participants who also met criteria for any Axis I PD were statistically significantly greater (p = 0.007, CI 95% 1.331-6.202) than those without an identified Axis I PD diagnosis (70.9% vs. 45.9%).

**Table 3:** **Prevalence of Co-Morbidity of Substance Use and Psychiatric Disorders**

	Tool used for diagnosis			Regression results for any disorder diagnosis	
**Prevalence of substance use disorders**	**Any psychiatric disorder**				
	**MINI (N=55)**	**Medical record (N=52)**	**No diagnosis by either measure (N=42)**	**OR**	**95% Confidence intervals**
Hazardous Drinking	25 (48.1%)	24 (46.2%)	17 (40.5%)	1.207	(0.557-2.615)
Opioid Use Disorder	15 (28.8%)	24 (46.2%)	13 (31.0%)	1.521	(0.682-3.392)
Cocaine Use Disorder	29 (55.8%)	28 (53.8%)	16 (38.1%)	1.715	(0.793-3.711)
	**Mood disorder**				
	**MINI (N=37)**	**Medical record (N=34)**	**No diagnosis by either measure (N=62)**	**OR**	**95% Confidence intervals**
Hazardous Drinking	19 (51.4%)	18 (52.9%)	22 (35.5%)	1.035	(0.467-2.293)
Opioid Use Disorder	15 (40.5%)	16 (47.1%)	21 (33.9%)	1.250	(0.558-2.802)
Cocaine Use Disorder	20 (54.1%)	19 (55.9%)	25 (40.3%)	0.857	(0.397-1.851)
	**Anxiety disorder**				
	**MINI (N=36)**	**Medical record (N=17)**	**No disorder by either measure (N=70)**	**OR**	**95% Confidence intervals**
Hazardous Drinking	15 (41.7%)	10 (58.8%)	27 (38.6%)	1.668	(0.774-3.595)
Opioid Use Disorder	17 (47.2%)	12 (70.6%)	20 (28.6%)	2.500*	**(1.150-5.435)**
Cocaine Use Disorder	18 (50%)	11 (64.7%)	27 (38.6%)	1.950	(0.917-4.145)
	**Thought disorder**				
	**MINI (N=13)**	**Medical record (N=16)**	**No diagnosis by either measure (N=91)**	**OR**	**95% Confidence intervals**
Hazardous Drinking	9 (69.2%)	8 (50%)	36 (40.9%)	0.593	(0.699-4.064)
Opioid Use Disorder	7 (53.8%)	6 (37.5%)	32 (35.2%)	1.352	(0.556-3.289)
Cocaine Use Disorder	9 (69.2%)	5 (31.3%)	42 (46.2%)	1.000	(0.417-2.397)

OR: Odds Ratio.

Hazardous Drinking defined by the Alcohol Use Disorders Identification Test (AUDIT), scores ≥8 for men, ≥4 for women (N=113).

Opioid and Cocaine Use Disorder defined by DSM-IV criteria via the Mini International Neuropsychiatric Interview (MINI) for abuse and dependence.

*p<0.05.

SUD co-morbidities among those who met criteria for an anxiety disorder using the MINI or the medical record were also high. Those with an anxiety disorder diagnosis by the MINI or medical record versus those without a diagnosis were statistically significantly more likely to meet criteria for opioid use disorder (p = 0.021, CI 95% 1.150-5.435). Elevated, but non-statistically significant differences were found for those with an anxiety disorder and having a concurrent cocaine or alcohol use disorders.

## Discussion

This study confirms high prevalence of both PDs and SUDs among HIV-infected prisoners who are transitioning to the community. It is imperative to accurately identify and treat these disorders before release since each can independently and negatively contribute to poor post-release treatment outcomes if left untreated.

The aim of the study was to compare differences in PD diagnoses when using a relatively quick and validated DSM-IV screening tool, the MINI (Lecrubier et al. [1997]; D. V. Sheehan et al. [1998a]; D. V. Sheehan et al. [1998b]), and compare findings to the medical record diagnosis of PLWHA during incarceration when they are mostly free from alcohol and drugs. Central to this study's findings is that it is challenging to make a PD diagnosis in the setting of active drug or alcohol use, but all of the study participants had been incarcerated for approximately 7 months and expect for unusual conditions, free from alcohol or drugs. This is also the first English-language study that examines the prevalence of Axis I PDs and SUDs among a group of HIV-infected prisoners who are transitioning to the community, and additionally compares the diagnoses made during incarceration. The hypothesis we formed was that the diagnoses would differ between the two measures and we found a fair level of agreement between the MINI and prison medical record for those with any PD, and fair to poor degrees of concordance between the three major categories explored in this analysis. The prevalence of mood disorders was similar based between the MINI and medical record (31.6% and 29.1%), but of those, only 28.6% were diagnosed using both measures. Anxiety disorder diagnoses were found to be different between the measures (MINI 31.0% vs. 14.7% medical record), however, and only 15.2% of individuals shared a diagnosis using both assessments. Similarly, the prevalence of thought disorders was 11.1% using the MINI vs. 13.7% using the medical record, with a mutual concordant diagnosis of only 11.5% from both measures. The prison medical record diagnoses matched moderately well with the MINI, thus suggesting that the prison system does a reasonable job in screening for PD among the inmates, although the kappa values suggest that significant improvements can be made for certain diagnoses.

Given the continuous concern of suicide among prisoners, screening questions that focus on measuring suicidal ideation, depression and psychosis and less on anxiety and thought disorders, may explain the higher rates of agreement for mood disorders versus the other disorders. Despite the staff and budget limitations of prisons, correctional facilities are able to improve and maintain the health of the inmates by directly administering the inmate's medications, providing increased medical attention, having access to support from staff and the general structure of prisons thus allowing inmates to live a more organized life. Extensive efforts have created a system to improve successful linkage of PLWHA upon release from prisons to appropriate community services, enabling continuity of care including the possible continuation of directly administered medications, for not only for cART but all medications, including psychiatric medications. Findings here, however, support the integration of HIV and psychiatric care for transitional care programs.

The prevalence of mood disorders by either measure in this study is high when compared to 6.8% in the general population (Reeves et al. [2011]), and some other correctional systems like in Texas where only 11.0% had reported diagnoses of major depressive, bipolar, non-schizophrenic psychotic disorders or schizophrenia (Baillargeon et al. [2009]). Similarly, 12.3% of U.S. adults report a lifetime diagnosis of an anxiety disorder, with higher prevalence among women and non-Hispanic white groups (Reeves et al. [2011]); much lower than the rate of 31.0% found in this analysis. Additionally, this analysis found an astounding prevalence of bipolar disorder (18.8%) compared to 1.7% nationally (Reeves et al. [2011]). Given the likely impulsivity of those with poorly controlled bipolar disorder and its contribution to SUDs, a targeted treatment plan to stabilize this population during incarceration and post-release to the community may improve retention in care, reduce relapse to drug and alcohol use and decrease HIV risk behaviors. Importantly, properly diagnosing bipolar disorder has important implications since treatments differ from other mood disorders. The prevalence in this study was also greater than those found in a different study of the Texas prisoners using the diagnosis of PDs solely from the medical record (Baillargeon et al. [2009]) where only 11.0% of 71,333 Texas inmates were diagnosed with a PD (Baillargeon et al. [2009]); and higher prevalence of PDs among HIV-infected when compared to their HIV negative counterparts (Baillargeon et al. [2003]). Overall there appears to be variance of reported PD diagnosis among prisoners in other studies, which may be due to the type of diagnostic screening tool utilized by correctional facilities line in Texas (Baillargeon et al. [2009]; Baillargeon et al. [2003]).

Although this study was specific to PLWHA, it is well known that PD are very common among all persons with the CJS including HIV uninfected persons. Thus likely improving accuracy of diagnosis of PD will improve care not only among PLWHA but also for all persons within the CJS and post-release (Brink [2005]). The MINI is not a gold standard over a trained psychiatric assessment of psychiatric disorders, but given the high concentration of PDs in the U.S. prisons, a standardized psychiatric diagnostic screening tool that can be administered quickly and accurately would enhance existing screening policies. Additionally, screening during every new admission could identify new symptoms of PD, allowing the facility to initiate treatment and arrange for continued treatment upon release. A single screening upon intake especially for those coming directly from the community where active drug or alcohol use, however, could complicate diagnostics and might lead to inaccurate diagnoses. Thus, innovative strategies that make acute PD diagnoses followed by assessments after periods of abstinence might better serve prisoners. For the duration of incarceration and after release, treatment of PD can increase cART adherence (Blower et al. [2000]), and reduce sexual risk behavior (Kalichman [2008]). Increased surveillance, treatment and continued care may also have implications for reduced PD symptoms thereby reducing relapse and reincarceation (Fu et al. [2013]). As a result, it may also improve the safety of the community and potentially reduce the risk of HIV transmission by reducing risk behaviors (Spaulding et al. [2002]).

Important in this analysis is the high prevalence of PD among HIV-infected prisoners transitioning to the community. Although standard PD screening algorithms in prisons identified many diagnoses, some were not identified and others were incorrectly diagnosed. While not all CJS settings may opt to use the MINI due to budgetary constraints (costs for the proprietary screening instrument or staff time required to administer it), brief and accurate validated screening and diagnostic instruments for PDs should be considered to consistently identify the PD to offer appropriate treatment and later refer to community resources upon release. Such validated PD screens developed specifically for CJS exist (Ford et al. [2009]; Steadman et al. [2005]) and can serve as a first stage in an efficient diagnostic process. Correctly identifying Axis I PDs and SUDs is of high importance for CJS-involved patients, while alternative ways to improve psychiatric treatment continue to be pursued. The calculus is heightened especially for PLWHA who also must transition effectively to the community. The transition from correctional facility to the community has been shown to be a troublesome time; and it has been shown that PD is a factor in reincarceration (Binswanger et al. [2007]; Baillargeon et al. [2010a]; Fu et al. [2013]). A focused treatment plan during their incarceration and after release is an important opportunity to reduce this risk that should include screening to identify PDs.

### Strengths and limitations

To our knowledge, this is the first study evaluating a specific DSM-IV disorder validated tool to evaluate prison-based psychiatric diagnoses among HIV-infected prisoners. By conducting this analysis we are able to begin to construct a single state system image of the current state of the mental health of HIV-infected prisoners, and find areas to improve comprehensive care. Limited within this evaluation, however, is the retrospective nature of this study among PLWHA in the CT CJS and a sample size that was not determined a priori to support this analysis, and limits the generalizability. Additionally, limited data were available to contribute to the different PD diagnoses including their association with criminal justice history. Nonetheless, it demonstrated high levels of PDs and SUDs in this population. The MINI 5.0 version used in this analysis did not include lifetime diagnosis criteria for all of the PDs, therefore the diagnosis of bipolar disorder becomes challenging given that there may be symptoms that have not manifested within the 12 months of pre-release assessment (potentially reducing the apparent prevalence). For those subjects incarcerated less than one year, there is also concern for confounding SUDs that may have masked or mimicked some symptoms as well. Additionally, a majority of the participants in the analysis have a history of prior incarcerations, and therefore it is likely that the diagnoses in the medical records may be older than the MINI diagnoses. In this context, the current sample diagnosed with a mood disorder had a co-morbid diagnosis of cocaine use disorder 54.1% and opioid use disorder 40.5%. Additional research is needed in this area to explore brief, validated screening instruments for PD to improve early identification, initiate appropriate psychiatric treatment and ensure continuity of care post-release in order to guide integrated care efforts for PLWHA with multiple co-occurring disorders as they transition to the community (Ford et al. [2009]; Steadman et al. [2005]).

## Conclusions

A consistent screening and assessment protocol for all DSM-IV PDs in the CJS may improve care in the facilities and with proper referrals, may improve retention in care upon release. Given that all PLWHA are now recommended to initiate cART, such strategies should have great impact on HIV treatment as prevention efforts (Montaner [2013]). Those with PDs increase the burden of care in prisons and the community upon release. Addressing the issue of identification and appropriate treatment for PDs in a controlled environment such as during incarceration can have implications within correctional facilities and later in the community. Given the recent release of DSM-V, this would be an ideal time for all correctional facilities and community supervision program to evaluate their current policies regarding PDs screening and treatment. Further research is needed in this area to examine the full implications of increased screening for PDs among PLWHA.

## Authors' contributions

FLA was responsible for the funding and the parent trial. AD, FLA and SAS were responsible for the analytic study design and AD and SAS drafted the manuscript. AD conducted the data analysis. FLA, MLP and RLT drafted selected sections of the manuscript. All authors provided feedback and approved the final manuscript.

## Authors’ original submitted files for images

Below are the links to the authors’ original submitted files for images. Authors’ original file for figure 1 Authors’ original file for figure 2 Authors’ original file for figure 3 Authors’ original file for figure 4

## Abbreviations

CJS: criminal justice system
PD: psychiatric disorder
SUD: substance use disorder
PLWHA: people living with HIV/AIDS
MINI: Mini International Neuropsychiatric Interview
cART: combination antiretroviral therapy
RCT: randomized controlled trial
DAART: directly administered antiretroviral treatment
SAT: self-administered treatment
CTDOC: Connecticut Department of Correction
AUDIT: Alcohol Use Disorder Identification Test
ASI: Addiction Severity Index
VL: HIV RNA viral load
CD4: CD4 lymphocyte counts
OCD: obsessive compulsive disorder
PTSD: post-traumatic stress disorder
GAD: generalized anxiety disorder
