# Supplementary figures and images for: A comparison of psychiatric diagnoses among HIV-infected prisoners receiving combination antiretroviral therapy and transitioning to the community
Source: Health Justice. 2014 Oct 29;2:11. doi: 10.1186/s40352-014-0011-1 (PMC4297667; doi:10.1186/s40352-014-0011-1)

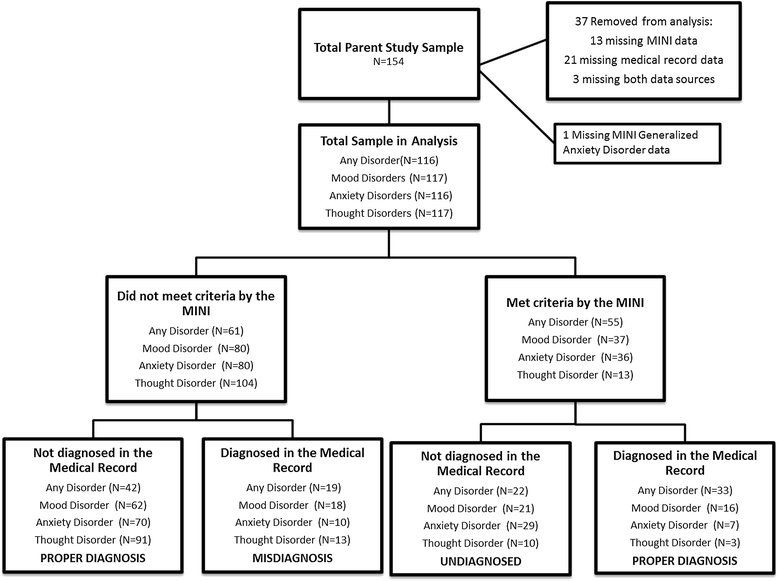

Supplement: Supplementary file 1 — Authors’ original file for figure 1 [file 40352_2014_11_MOESM1_ESM.gif]
