# Supplementary material for: A comparison of psychiatric diagnoses among HIV-infected prisoners receiving combination antiretroviral therapy and transitioning to the community
Source: Health Justice. 2014 Oct 29;2:11. doi: 10.1186/s40352-014-0011-1 (PMC4297667; doi:10.1186/s40352-014-0011-1)
Supplement: Supplementary file 2 — Authors’ original file for figure 2 [file 40352_2014_11_MOESM2_ESM.docx]

Table 1. Baseline Characteristics

| **Baseline Characteristics** | **Total Sample** |
| --- | --- |
|  | **(N=117)** |
| Gender |  |
| Male | 96 (82.1%) |
| Female | 21 (17.9%) |
| Mean Age, years (SD) | 45.4 (±6.9) |
| Ethnicity |  |
| White | 16 (13.7%) |
| African-American | 63 (53.8%) |
| Hispanic | 38 (32.5%) |
| Anticipated Housing |  |
| Unstable Housing | 60 (51.3%) |
| Homeless | 29 (24.8%) |
| Stable Housing | 21 (17.9%) |
| Opioid Use Disorder* | 43 (36.8%) |
| Cocaine Use Disorder* | 54 (46.2%) |
| Hazardous Drinking (AUDIT**) | 50 (42.7%) |
| Viral Load (Baseline) |  |
| < 400 copies/mL | 92 (78.6%) |
| < 50 copies/mL | 66 (56.4%) |
| Mean Log HIV-1 RNA Level, cells/mL (SD) | 2.300 (±1.02) |
| CD4+ lymphocytes count, cells/mL (SD) | 403.61 (±244.4) |
| Addiction Severity Index Composite Scores | |
| Psychiatric Composite Score (n=112) |  |
| Mean Severity Score (SD) | 0.254 (±0.25) |
| High Severity (>.22) | 52 (46.4%) |
| Drug Use Composite Score (n=113) |  |
| Mean Severity Score (SD) | 0.085 (±0.08) |
| High Severity (>.16) | 15 (13.3%) |
| Alcohol Use Composite Score (n=112) |  |
| Mean Severity Score (SD) | 0.055 (±0.11) |
| High Severity (>.17) | 8 (7.1%) |
| Prescribed Psychiatric Medications | 35 (29.9%) |
| Anti-Depressants | 22 (18.8%) |
| Anti-Psychotics | 2 (1.7%) |
| Mood Stabilizers | 1 (0.9%) |
| Multiple Medications | 16 (13.7%) |
| Any Psychiatric Disorder (n=116)* | 55 (47.4%) |
| Any Mood Disorder* | 37 (31.6%) |
| Any Anxiety Disorder (n=116)* | 36 (31.0%) |
| Any Thought Disorder* | 13 (11.1%) |

SD=standard deviation

*Criteria for diagnosis defined by the Mini International Neuropsychiatric Interview (MINI). Substance use disorders combine abuse and dependence diagnosis criteria.

**Hazardous Drinking defined by the Alcohol Use Disorders Identification Test (AUDIT), scores >8 for men, >4 for women.
