# Supplementary material for: A comparison of psychiatric diagnoses among HIV-infected prisoners receiving combination antiretroviral therapy and transitioning to the community
Source: Health Justice. 2014 Oct 29;2:11. doi: 10.1186/s40352-014-0011-1 (PMC4297667; doi:10.1186/s40352-014-0011-1)
Supplement: Supplementary file 3 — Authors’ original file for figure 3 [file 40352_2014_11_MOESM3_ESM.docx]

Table 2: Diagnoses Frequencies by Measure and Kappa Values

| **Psychiatric Disorder (N=117)** | | **MINI Diagnosis** | **Medical Record Diagnosis** | **No Diagnosis by Either Measures** | **Diagnoses Captured by both Measures** | **Kappa Level of Agreement** |
| --- | --- | --- | --- | --- | --- | --- |
| **Any Diagnosis (n=116)** | | 55 (47.4%) | 52 (44.8%) | 42 (36.2%) | 33 (28.4%) | 0.294 Fair |
| **Mood Disorder** | | 37 (31.6%) | 34 (29.1%) | 62 (53.0%) | 16 (29.1%) | 0.212 Fair |
|  | Major Depressive Disorder | 15 (12.8%) | 26 (22.2%) | 82 (70.1%) | 6 (24%) | 0.155 Poor |
|  | Bipolar Disorder | 22 (18.8%) | 15 (12.8%) | 82 (70.1%) | 2 (5.7%) | -0.052 Poor |
| **Anxiety Disorder (n=116)** | | 36 (31.0%) | 17 (14.7%) | 70 (59.8%) | 7 (15.2%) | 0.081 Poor |
|  | Panic Disorder | 17 (14.7%) | 0 (0.0%) | 99 (85.3%) | 0 (0.0%) | 0.000 Poor |
|  | Obsessive Compulsive  Disorder | 14 (12.0%) | 0 (0.0%) | 103 (88.0%) | 0 (0.0%) | 0.000 Poor |
|  | Post-Traumatic Stress  Disorder | 8 (6.9%) | 13 (11.2%) | 97 (82.9%) | 1 (5%) | 0.012 Poor |
|  | Generalized Anxiety | 13 (11.2%) | 5 (4.3%) | 99 (85.3%) | 1 (5.9%) | 0.052 Poor |
| **Thought Disorder** | | 13 (11.1%) | 16 (13.7%) | 91 (77.8%) | 3 (11.5%) | 0.096 Poor |
