# Supplementary material for: A comparison of psychiatric diagnoses among HIV-infected prisoners receiving combination antiretroviral therapy and transitioning to the community
Source: Health Justice. 2014 Oct 29;2:11. doi: 10.1186/s40352-014-0011-1 (PMC4297667; doi:10.1186/s40352-014-0011-1)
Supplement: Supplementary file 4 — Authors’ original file for figure 4 [file 40352_2014_11_MOESM4_ESM.docx]

Table 3: Prevalence of Co-Morbidity of Substance Use and Psychiatric Disorders

|  | **Tool Used for Diagnosis** | | | | | | | **Regression Results for any Disorder Diagnosis** | |
| --- | --- | --- | --- | --- | --- | --- | --- | --- | --- |
| **Prevalence of Substance Use Disorders** | **Any Psychiatric Disorder** | | | | | | | | |
|  | **MINI (N=55)** | **Medical Record (N=52)** | | | **No Diagnosis by Either Measure (N=42)** | | **OR** | | **95% Confidence Intervals** |
| Hazardous Drinking | 25 (48.1%) | 24 (46.2%) | | | 17 (40.5%) | | 1.207 | | (0.557-2.615) |
| Opioid Use Disorder | 15 (28.8%) | 24 (46.2%) | | | 13 (31.0%) | | 1.521 | | (0.682-3.392) |
| Cocaine Use Disorder | 29 (55.8%) | 28 (53.8%) | | | 16 (38.1%) | | 1.715 | | (0.793-3.711) |
|  | **Mood Disorder** | | | | | | | | |
|  | **MINI (N=37)** | **Medical Record (N=34)** | | | **No Diagnosis by Either Measure (N=62)** | | **OR** | | **95% Confidence Intervals** |
| Hazardous Drinking | 19 (51.4%) | 18 (52.9%) | | | 22 (35.5%) | | 1.035 | | (0.467-2.293) |
| Opioid Use Disorder | 15 (40.5%) | 16 (47.1%) | | | 21 (33.9%) | | 1.250 | | (0.558-2.802) |
| Cocaine Use Disorder | 20 (54.1%) | 19 (55.9%) | | | 25 (40.3%) | | 0.857 | | (0.397-1.851) |
|  | **Anxiety Disorder** | | | | | | | | |
|  | **MINI (N=36)** | | **Medical Record (N=17)** | | **No Disorder by Either Measure (N=70)** | **OR** | | | **95% Confidence Intervals** |
| Hazardous Drinking | 15 (41.7%) | | 10 (58.8%) | | 27 (38.6%) | 1.668 | | | (0.774-3.595) |
| Opioid Use Disorder | 17 (47.2%) | | 12 (70.6%) | | 20 (28.6%) | **2.500*** | | | **(1.150-5.435)** |
| Cocaine Use Disorder | 18 (50%) | | 11 (64.7%) | | 27 (38.6%) | 1.950 | | | (0.917-4.145) |
|  | **Thought Disorder** | | | | | | | | |
|  | **MINI (N=13)** | | **Medical Record (N=16)** | **No Diagnosis by Either Measure (N=91)** | | | **OR** | | **95% Confidence Intervals** |
| Hazardous Drinking | 9 (69.2%) | | 8 (50%) | 36 (40.9%) | | | 0.593 | | (0.699-4.064) |
| Opioid Use Disorder | 7 (53.8%) | | 6 (37.5%) | 32 (35.2%) | | | 1.352 | | (0.556-3.289) |
| Cocaine Use Disorder | 9 (69.2%) | | 5 (31.3%) | 42 (46.2%) | | | 1.000 | | (0.417-2.397) |

OR: Odds Ratio

Hazardous Drinking defined by the Alcohol Use Disorders Identification Test (AUDIT), scores >8 for men, >4 for women (N=113).

Opioid and Cocaine Use Disorder defined by DSM-IV criteria via the Mini International Neuropsychiatric Interview (MINI) for abuse and dependence.

* p<0.05
